# Programming of stress-related behavior and epigenetic neural gene regulation in mice offspring through maternal exposure to predator odor

**DOI:** 10.3389/fnbeh.2015.00145

**Published:** 2015-06-01

**Authors:** Sophie St-Cyr, Patrick O. McGowan

**Affiliations:** ^1^Department of Biological Sciences and Center for Environmental Epigenetics and Development, Department of Cell and Systems Biology, University of TorontoToronto, ON, Canada; ^2^Department of Psychology, University of TorontoToronto, ON, Canada; ^3^Department of Physiology, University of TorontoToronto, ON, Canada

**Keywords:** predator odor, maternal programming, *BDNF*, epigenetic, *CRHR1*, stress, hippocampus, amygdala

## Abstract

Perinatal stress mediated through the mother can lead to long-term alterations in stress-related phenotypes in offspring. The capacity for adaptation to adversity in early life depends in part on the life history of the animal. This study was designed to examine the behavioral and neural response in adult offspring to prenatal exposure to predator odor: an ethologically-relevant psychological stressor. Pregnant mice were exposed daily to predator odors or distilled water control over the second half of the pregnancy. Predator odor exposure lead to a transient decrease in maternal care in the mothers. As adults, the offspring of predator odor-exposed mothers showed increased anti-predator behavior, a predator-odor induced decrease in activity and, in female offspring, an increased corticosterone (CORT) response to predator odor exposure. We found a highly specific response among stress-related genes within limbic brain regions. Transcript abundance of Corticotropin-releasing hormone receptor 1 (*CRHR1*) was elevated in the amygdala in adult female offspring of predator odor-exposed mothers. In the hippocampus of adult female offspring, decreased Brain-derived neurotrophic factor (*BDNF*) transcript abundance was correlated with a site-specific decrease in DNA methylation in *Bdnf* exon *IV*, indicating the potential contribution of this epigenetic mechanism to maternal programming by maternal predator odor exposure. These data indicate that maternal predator odor exposure alone is sufficient to induce an altered stress-related phenotype in adulthood, with implications for anti-predator behavior in offspring.

## Introduction

The transmission of information about a stressful prenatal environment from a mother to her offspring is thought to be highly relevant in optimally preparing the offspring for the environment they will face later in life (Meaney, 2001; Welberg and Seckl, 2001). This form of early life “programming”, mediated prenatally through the maternal physiology or postnatally via changes in maternal behavior, can have impacts on the offspring phenotype that persist throughout the lifespan. Accumulating evidence indicates that these effects are mediated in part by epigenetic changes (Weaver et al., 2004; Mueller and Bale, 2008), defined as modifications to chromatin or DNA that collectively establish and propagate differential gene expression without a change in DNA sequence (Roth et al., 2009). In prey animals such as rodents, predator odor is a stressor that is ecologically relevant, unconditioned and psychogenic. Animals exposed to predator odor show a wide range of impacts, including a long-term increase in anxiety behaviors, changes in activity levels, increased levels of circulating glucocorticoids (GCs), decreased body weight and altered transcript abundance of brain-derived neurotrophic factor (*BDNF*; Adamec et al., 2004; Zoladz et al., 2008; Bazak et al., 2009).

The impacts of early life exposure to live predators or to cues that indicate the threat of predation have been examined in a range of organisms in natural populations, semi-natural settings, and in typical laboratory settings. The response to predator threat in early life is largely species-specific, but is often associated with increased defensive behaviors and stress reactivity across the lifespan. For example, prenatal exposure to a predator cues leads to the production of protective helmets in *Daphnia* (Agrawal et al., 1999), increased egg size with higher corticosterone (CORT) concentrations and metabolism in the threespine stickleback (Giesing et al., 2011), and lower birth weight, shorter body length, increased stress hormone levels and stress reactivity in snowshoe hares in natural environments with a high density of predators (Sheriff et al., 2009). Behavioral responses linked to prenatal predator exposure include increased hiding behavior linked to increased duration of survival in the presence of a live predator in adult crickets, impaired anti-predator responses and a higher susceptibility to predation in threespine stickleback (Giesing et al., 2011; McGhee et al., 2012), and increased locomotor activity in the common lizard (Bestion et al., 2014).

Very little research has examined the effect of early life predator stress on phenotype in laboratory rodents. In rats, the two existing studies examining the effects of predator exposure during gestation have reported a link between maternal exposure and predisposition to seizures in offspring, associated with altered hippocampal plasticity (Ahmadzadeh et al., 2011; Saboory et al., 2011). In mice and rats, exposing dams to predator odor in the first week of postnatal life or from birth to weaning increases levels of licking and nursing of offspring (McLeod et al., 2007; Coutellier and Würbel, 2009; Mashoodh et al., 2009). Rat offspring exposed to predator odor during early postnatal life (post-natal day 0 or 3) also show altered anxiety-like behaviors in the Open Field task and increased avoidance behavior during predator odor exposure in adulthood (Mashoodh et al., 2009). These studies suggest that direct exposure to predator odor during the lactational period of maternal care may partly mediate the effects of direct exposure to predator odor during early postnatal life. To our knowledge, the influence of prenatal exposure to predator odor in rodents on the stress-related phenotype of adult offspring has not been examined.

We exposed pregnant C57BL6 mouse dams to predator odor during the second half of their pregnancy, a primary period of hypothalamic-pituitary-adrenal (HPA) axis development in offspring, and evaluated its impact on offspring growth as well as behavior and neural gene regulation in adulthood. We hypothesized that maternal exposure to this ecologically relevant and psychological stressor would decrease body weight and growth, increase in anti-predatory behavior, enhance the endocrine response to stress and alter related gene regulation in limbic brain areas in offspring. We found that mice from predator odor exposed dams exhibited behaviors consistent with heightened anxiety and an increased CORT response to predator odor exposure. We also found sex-specific alterations in the expression of stress-related genes in the amygdala and hippocampus, including CRHR1 and the *BDNF* gene, where decreased expression was associated with decreased exonic DNA methylation among adult female offspring from predator odor-exposed dams.

## Materials and Methods

### Animal Housing and Breeding

Twenty-eight female and 14 male adult C57BL/6 mice were obtained from Charles River Canada (St. Constant, QC). The mice were housed in same-sexed groups (3–5 per cage) and maintained on a 12:12 h light-dark cycle (lights on at 7:00 am) with *ad libitum* access to food and water. All experimental protocols were approved and conform to the Local Animal Care Committee at the University of Toronto in Scarborough regulatory standards and were in accordance with the guidelines of the Canadian Council on Animal Care.

For breeding, two females were housed with one male between 9:00 am and 5:00 pm. Females were then checked for sperm plugs indicating gestational day 0. Pregnant females were singly housed and weighed every other day throughout the pregnancy. During initial mating, five females exposed to 19.4 μl of pure 2, 3, 5-Trimethyl-3-thiazolin (TMT; Wallace and Rosen, 2000) failed to reach parturition, therefore dosing was reduced for the remaining predator odor-exposed females (see below). Of the 23 remaining mated females, two failed to get pregnant and two control litters were excluded due to low pup numbers. A total of 19 mated females gave birth to 11 litters from predator odor-exposed dams and eight control litters.

### Predator Odor Exposure in Pregnant Dams

**Figure 1:**
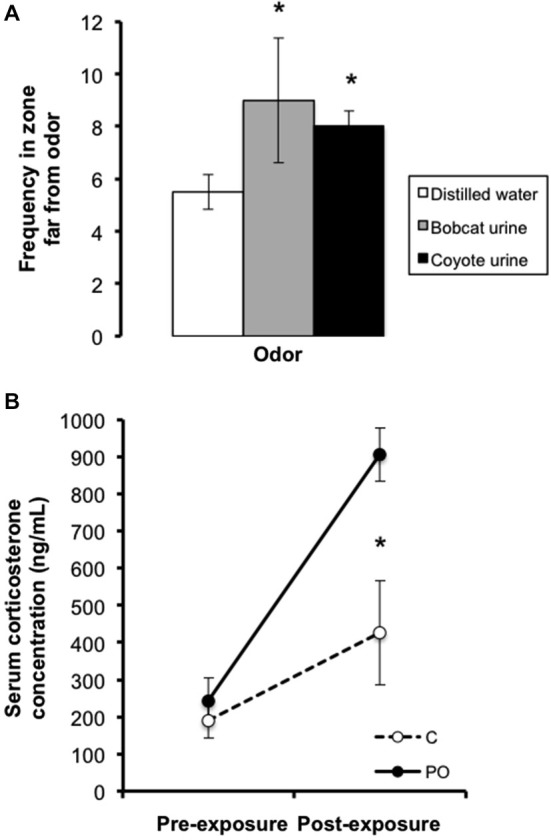
**Preliminary testing in adult females showing (A) bobcat (3 mL) and coyote (5 mL) urine elicited avoidance of the predator odor source relative to distilled water control and (B) elevated basal CORT levels after a week of unpredictable 1 h daily predator odor exposure**. Data are average ± SEM. Predator Odor (PO); Control (C). *P ≤ 0.05, effect of predator odor exposure.

Prior to predator odor exposure sessions, pregnant dams were habituated to a room with a fume hood for five consecutive days prior breeding. From gestational day 11–18, dams were introduced to a novel cage (15 × 33 cm) and presented with liquid odors on cotton balls sealed in a petri dish with holes, used to avoid direct contact with the odorants. Dams in the predator odor condition were exposed once per day for 1 h during the light cycle for a total of three times to 3 mL of bobcat urine, three times to 5 mL of coyote urine^1^, and two times to 100 μl of a 1:5000 solution of TMT dissolved in mineral oil (Contech enterprises #300000368, Mueller and Bale, 2006) in a randomized manner. Each exposure lasted for 1 h, based on previously published protocols (Belzung et al., 2001; Mueller and Bale, 2007; Mashoodh et al., 2009). Control dams were exposed to the same regimen at the same time each day, except that distilled water was used instead of the odorants. In preliminary testing of the behavioral response to predator odor in adult females, a cage was divided into three zones: far, neutral and near a predator odor source, and the animal’s location was measured once per minute over a 15 min trial. Bobcat and coyote urine elicited anti-predator behaviors, as females exposed to predator odor (*n* = 4 per odor) stayed in the “far” zone more frequently relative to females exposed to distilled water control (*n* = 16; Figure 1A). As well, in additional preliminary testing in a separate group of animals, females showed elevated basal CORT levels after 1 week of exposures to predator odors compared to distilled water control using the same exposure regimen as above (*n* = 4 per group; Figure 1B). TMT is a component of fox feces that has been widely used in predator odor stress exposure studies (Wallace and Rosen, 2000; Mueller and Bale, 2006; Takahashi et al., 2008).

### Maternal Behaviors in Dams and Offspring Morphological Measures

The maternal behavior of predator odor exposed and control dams was examined between postnatal day (PN) 1–6. At PN0, litters were culled to a maximum of six pups. Maternal behaviors were video recorded for 1 h, six times a day throughout the animal’s subjective light phase (7:00 am, 11:00 am, 3:00 pm) and dark phase (7:00 pm, 11:00 pm, 3:00 am; Champagne et al., 2007). Focal maternal behaviors including licking, nursing (high-crouch, low-crouch, supine), hovering, nesting, retrieving and time on the nest were coded using Observer XT 8.5 (Noldus, USA) every 3 min for a given observation period (20 observations/period × 6 periods = 120 observations/mother/day). The percentage of maternal behaviors was calculated by dividing the frequency observed by the number of observations made per day.

The morphology of the offspring was examined from birth to adulthood. Sex, body weight and body length from the tip of the snout to the base of the tail were measured on PN0 using a scale (to the nearest 0.1 g) and vernier caliper (to the nearest 0.1 mm), respectively. Offspring were left undisturbed except for a weekly weighing and measuring until PN21, when they were housed in same-sex groups of up to five individuals. Measurements of body length continued up to PN56 during the weekly cage changes while body weights were measured weekly up to PN56, and then on PN90 and PN140.

### Predator Odor Avoidance Test and Corticosterone Response to Predator Odor Exposure in Adult Offspring

The behavioral response to predator odor exposure and the CORT response to predator odor exposure were evaluated in adult offspring (PN90). Mice were handled (3 min/day) for seven consecutive days prior to testing. Male and female adult offspring (1–2/sex/litter) were used in the predator odor avoidance test and for CORT radioimmunoassays with predator odor exposure (C-M *n* = 13, C-F *n* = 8, PO-M *n* = 13, PO-F *n* = 13).

For the predator odor avoidance test, mice were habituated to a procedure room with a fume hood for 3 h on the day of testing. The predator odor exposure took place in a transparent arena (45 × 24 cm) placed in the fume hood. The mice were exposed to 3 mL of bobcat urine at one end of the arena and to a circular shelter (10 cm diameter) at the other end of the arena. Predator odor avoidance, quantified as the average distance from the predator odor over each 10 min of the 30 min trial, and the total distance travelled during each 10 min of the trial were recorded. The test was video recorded and behavior was tracked in an automated fashion using EthoVision XT10 (Noldus, USA).

The CORT response to predator odor in offspring was measured a minimum of 4 days after behavioral testing. To measure the CORT response to predator odor exposure, mice were habituated to a procedure room with a fume hood for 3 h and then hand-restrained in a loosely fitting towel. Blood was withdrawn from a small nick in the tail for baseline CORT measurement (0 min) within 3 min. TMT odor was used to assess the CORT response to predator odor exposure because mice were exposed to bobcat urine previously. Mice were exposed to 100 μl of a 1:5000 TMT solution in mineral oil on a cotton ball sealed in a petri dish with holes placed in a transparent cage (15 × 33 cm) inside the fume hood for 60 min, and a second blood sample was collected to measure levels of CORT immediately following this exposure (60 min). Mice were then returned to their home cage and left undisturbed for 60 min, after which a third blood sample was collected to measure recovery CORT levels (120 min). Blood was kept on ice for at least 30 min before being centrifuged at 4,000 rpm at 4°C for 20 min. Serum was then extracted and stored at −80°C. The amount of CORT present in the serum was determined using a radioimmunoassay kit with 125I-labeled anti-corticosterone antibody (MP Biomedicals Inc., CA, USA: sensitivity 7.7 ng/mL, intra-assay coefficient of variation 10.3%). Where two animals of the same sex and litter were tested, the samples were pooled in equal amounts. The final sample sizes were C-M *n* = 8, C-F *n* = 6, PO-M *n* = 8 and PO-F *n* = 8 for each time point and each sample was assayed in duplicate.

### Tissue Preparation and Nucleotide Extraction

A separate cohort of adult male and female offspring (*n* = 6 per prenatal stress group, 1 mouse/sex/litter) was used for nucleotide extraction. Mice were sacrificed (PN130 ± 10) by CO_2_ inhalation followed by decapitation. Whole brains were flash frozen in isopentane on dry ice and stored at −80°C. The entire hippocampus (Bregma −1.22 mm to +3.08 mm) and amygdala (Bregma −0.58 mm to −1.94 mm) were dissected from 50 μm microsections with a Research Cryostat Leica CM3050 S (Leica Biosystems) using stereotaxic coordinates (Franklin and Paxinos, 1997). RNA and DNA were extracted using a combination of Trizol/chloroform and the Allprep kit (Qiagen). RNA was converted to cDNA (Applied BioSystems High Capacity cDNA Conversion Kit). Nucleotide quantification and purity were assessed with a spectrophotometer (Nanodrop ND-2000C, Thermo Scientific).

### Gene Expression Analysis by Quantitative Real-Time Reverse Transcriptase-Polymerase Chain Reaction (qRT-PCR)

Gene expression levels in the hippocampus and amygdala were quantified using a StepOne Plus real-time thermocycler and Fast SYBR Green PCR master mix (Applied Biosystems, Life Technologies, Carlsbad, CA, USA). The expression of 11 transcripts of interest was quantified: *BDNF*, glucocorticoid receptor (*GR*), mineralocorticoid receptor (*MR*), FK506 binding protein 5 (*FKBP5*), *CRH*, *CRHR1*, *CRHR2*. These genes have known roles in stress-related responses. The expression of four housekeeping genes (*GAPDH*, *ACTB*, *YWHAZ*, and *18S*) was also assessed. Table 1 shows the primers used in this study, which were designed using sequence information from GeneBank at the National Center for Biotechnology Information (NCBI)^2^ and Ensembl^3^. Gene expression was quantified relative to *YWHAZ*, the gene showing the least variance between the prenatal stressed and control offspring according to a previously published algorithm (NormqPCR R script, available at: http://www.bioconductor.org/packages/release/bioc/html/NormqPCR.html). A standard curve was generated with 11 serial dilutions of a mixture of cDNA from all 24 offspring (6/sex/prenatal stress group). Quantification was carried out in triplicate, and the average relative expression for each sample was used for analysis.

**Table 1:** **Primer sequences**.

Gene	Forward primer (5′-3′)	Reverse primer (5′-3′)
*YWHAZ*	TTGAGCAGAAGACGGAAGGT	GAAGCATTGGGGATCAAGAA
*GR*	AACTGGAATAGGTGCCAAGG	GAGGAGAACTCACATCTGGT
*MR*	GAAGAGCCCCTCTGTTTGCAG	TCCTTGAGTGATGGGACTGTG
*FKBP5*	CGGAAAGGCGAGGGATACTC	TTCCCCAACAACGAACACCA
*CRH*	GCAGCCCTTGAATTTCTTGCA	TCTTCACCCATGCGGATCAG
*CRHR1*	CGCAAGTGGATGTTCGTCT	GGGGCCCTGGTAGATGTAGT
*CRHR2*	CCCCTGTGGACACTTTTGGA	AGGTCGTGTTGCAGTAGGTG
*BDNF*	AGGTTCGAGAGGTCTGACGA	ATGTTTGCGGCATCCAGGTA
BDNFmO	AGATGTATTATTTTAAATGCGCGGA	ACTTCCCAACAAACCAAACGA
BDNFmN	AGAGTGTTTATTTAGAGGTAGAGGAGGTAT	AACCCATCCCCAAAATTCTAAACTCT
BDNFseq	TTTGTTTAGATTAAATGGA	

*YWHAZ: 14-3-3 protein zeta/delta, GR: Glucocorticoid receptor, MR: Mineralocorticoid receptor, FKBP5: FK506 binding protein 5, CRH: Corticotropin-releasing hormone, CRHR1: Corticotropin-releasing hormone receptor 1, CRHR2: Corticotropin-releasing hormone receptor 2, BDNF: Brain-derived neurotrophic factor, BDNFmO: BDNF methylation Outside, BDNFmN: BDNF methylation Nested, BDNFseq: BDNF sequencing primer*.

### DNA Methylation Analysis by Bisulfite Pyrosequencing

DNA methylation of the *Bdnf* exon *IV* gene was examined by bisulfite pyrosequencing for six female offspring from predator odor-exposed dams and six control female offspring. Bisulfite conversion of 2 μg of DNA per sample was performed using the EpiTect Bisulfite Kit (Qiagen). Biotinylated PCR products were obtained using outside and nested PCR primers targeting four CpG sites within *Bdnf* exon *IV* proximal to the transcription initiation site (Table 1; Figure 6A; Lubin et al., 2008). Pyrosequencing was performed with a Pyromark Q106 ID pyrosequencer, and CpG methylation levels were quantified using Pyromark Q-CpG 1.0.9 software. Analysis of 2–4 replicates was carried out for each sample. Replicates with a coefficient of variation greater than 30% were considered outliers and removed from analysis (*n* = 1 offspring from a predator exposed dam and *n* = 1 control offspring).

### Statistical Analyses

Statistical analyses were carried out using SPSS (IBM). Data for maternal behaviors were analyzed by 2 × 6 predator odor by day mix-model repeated measures analysis of variance (ANOVA). 2 (maternal predator odor exposure) × 2 (sex) × 3 (time) mixed-model repeated measures ANOVA followed by *post hoc* analyses were used to examine the behavioral and CORT response to predator odor exposure in adult offspring. 2 (maternal predator odor exposure) × 2 (sex) factorial ANOVA followed by *post hoc* analyses were used for gene expression analysis. A 2 (maternal predator odor exposure) × 4 (CpG site) factorial ANOVA was used to examine DNA methylation levels between groups. Student’s *t*-tests were used for pairwise CpG methylation level comparisons between offspring from predator odor-exposed dams and controls. Correlational analysis was used to assess the relationship between *BDNF* gene expression levels and *BDNF* DNA methylation levels in the hippocampus as well as CRHR1 expression and the CORT response to predator odor exposure. Effects were considered statistically significant at *P* ≤ 0.05 and some non-significant trends at *P* ≤ 0.10 are reported. *Post hoc* analyses were performed using protected least significant difference (PLSD) testing.

## Results

### Maternal Behavior

**Figure 2:**
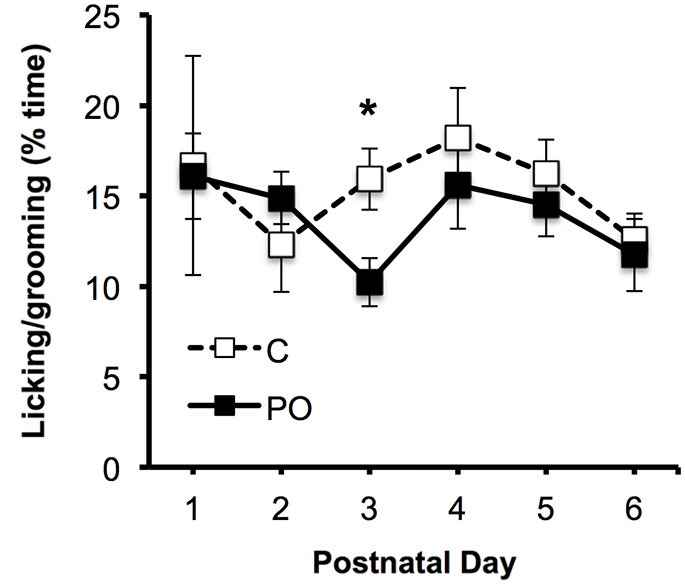
**Maternal behavior in predator odor exposed (PO) and control (C) dams, showing the proportion of time spent licking the pups**. Data are average ± SEM. *P ≤ 0.05, effect of predator odor exposure.

Maternal behavior was examined in predator-odor exposed dams and control dams between PN1–6. There was a significant interaction between postnatal day and predator odor exposure in the percent time that dams spent licking and grooming their offspring (*F*_(5,75)_ = 3.04, *P* = 0.02), with control dams showing increased licking on PN3 compared to predator odor-exposed dams (*P* = 0.02; Figure 2). There was no effect of maternal predator odor exposure on the other measures of maternal behavior examined, the length of pregnancy, litter size, offspring sex ratios, offspring body weight, offspring body length or offspring sex ratios (*P*’s > 0.05).

### Behavioral and Corticosterone Response to Predator Odor Exposure in Adult Offspring

**Figure 3:**
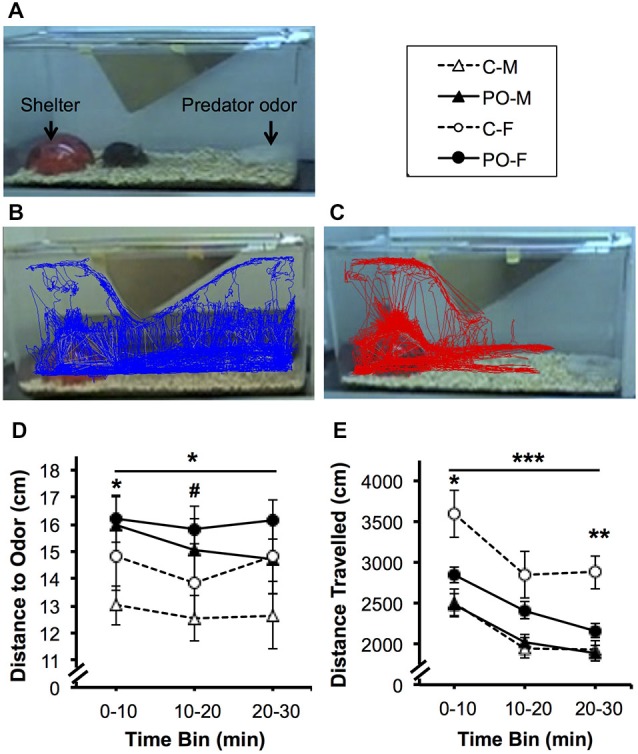
**Adult offspring from dams exposed to predator odor during pregnancy (PO) show increased avoidance and decreased predator-odor associated activity compared to control (C) adult offspring**. M, male; F, female. **(A)** Predator odor exposure setup. Representative 30-min. tracing of locomotor activity from **(B)** control and **(C)** maternal predator odor-exposed adult offspring. **(D)** Average distance to the predator odor and **(E)** overall distance travelled. Data are average ± SEM. Bars, *P ≤ 0.05, ***P ≤ 0.001, main effect of maternal predator odor exposure; ^#^*P* ≤ 0.10, *P ≤ 0.05, **P ≤ 0.01 *post hoc* comparison within sex.

When the offspring reached adulthood, they were tested for their response to predator odor exposure. Overall, the adult offspring of dams exposed to predator odor showed increased avoidance behavior when exposed to bobcat urine (Figures 3A–C). The offspring of predator odor-exposed dams stayed farther away from the predator odor compared to control offspring overall during the 30 min trial (*F*_(1,44)_ = 4.29, *P* = 0.04; Figure 3D). Male offspring from predator odor-exposed dams stayed farther from the source of the predator odor on average compared to male control offspring (*P* < 0.05 at 0–10 and *P* < 0.09 at 10–20 min). In addition, when exposed to predator odor in adulthood, offspring from predator-odor exposed dams were less active than control offspring, traveling a shorter distance over the course of the trial (*F*_(1,44)_ = 4.38, *P* = 0.04; Figure 3E). The effect of dams’ predator odor exposure on offspring locomotor activity varied over time (*F*_(2,88)_ = 3.13, *P* = 0.05, maternal predator odor exposure × time interaction) and as a function of sex (*F*_(1,44)_ = 4.45, *P* = 0.04). Female offspring from predator odor-exposed dams also traveled less distance than control female offspring in the first 10 min and last 10 min of the trial (*P*’s < 0.05 for PO females vs. control females at 0–10 and 20–30 min).

**Figure 4:**
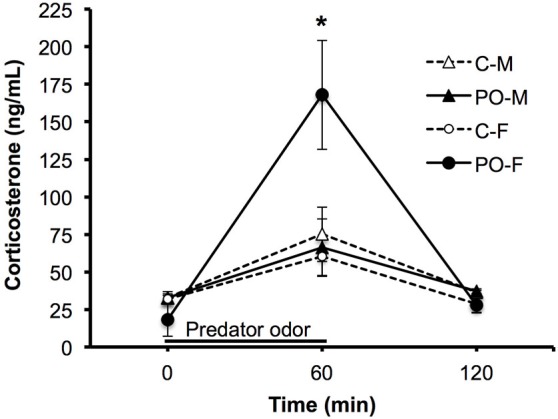
**Adult offspring from dams exposed to predator odor during pregnancy (PO) show an altered predator odor-challenged levels of CORT to a predator odor compared to control (C) adult offspring**. M, male; F, female. The black line shows the duration of the predator odor exposure. Data are average ± SEM. *P ≤ 0.05, *post hoc* comparison within female offspring.

The same subjects were tested for their CORT response to TMT predator odor exposure a minimum of 4 days after the completion of behavioral testing. CORT levels were examined at baseline (0 min), after an hour exposure to predator odor (60 min) and an hour after exposure to assess return to baseline (120 min). Overall, all adult offspring showed a significant rise in CORT levels in response to predator odor exposure and a significant decrease in CORT levels between the 60 min and 120 min time points (*F*_(2,52)_ = 24.31, *P* < 0.001; Figure 4). The CORT response over time differed between offspring from dams exposed to predator odor and control offspring (*F*_(2,52)_ = 4.55, *P* = 0.02, time × maternal predator odor exposure interaction). The CORT response over time also differed between males and females (*F*_(2,52)_ = 4.24, *P* = 0.02, time × sex interaction). There was a significant three-way interaction between all three variables (*F*_(2,52)_ = 6.23, *P* = 0.004, time × sex × maternal predator odor exposure interaction). Female offspring from predator odor-exposed dams showed significantly higher CORT levels immediately after exposure to the predator odor compared to female control offspring (*P* = 0.03).

### Gene Expression Analysis in Adult Offspring

**Figure 5:**
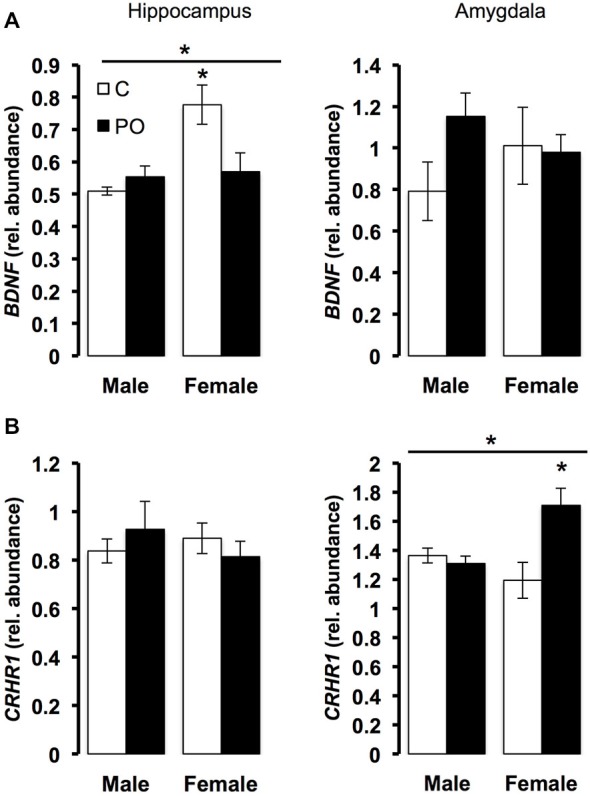
**Prenatal exposure to predator odor leads to differences in transcript abundance in the hippocampus and amygdala of the adult offspring**. C, control; PO, offspring of predator odor exposed dams. Respectively, hippocampal and amygdala *BDNF* **(A)** and *CRHR1* **(B)** transcript relative abundance corrected using *YWHAZ* transcript level. Data are average ± SEM. Bars, *P ≤ 0.05, main effect of maternal predator odor exposure; *P ≤ 0.05, *post hoc* comparison within sex.

The expression of stress-related genes in the hippocampus and amygdala was measured in adult offspring from predator odor exposed dams and controls. In the hippocampus, there was a significant interaction between maternal predator odor condition and offspring sex for *BDNF* expression (*F*_(1,20)_ = 7.70, *P* = 0.01). Female offspring showed greater expression of *BDNF* compared to male offspring (*F*_(1,20)_ = 9.58, *P* < 0.01), an effect due to significantly greater *BDNF* among control female offspring compared to female offspring from predator odor-exposed dams (*P* = 0.03; Figure 5A). There were no differences in the expression of *CRHR1* in the hippocampus. In the amygdala, offspring from predator odor-exposed dams showed higher levels of expression of *CRHR1* compared to control offspring (*F*_(1,20)_ = 6.29, *P* = 0.02), and *CRHR1* expression levels varied as a function of maternal predator odor condition and offspring sex (*F*_(1,20)_ = 9.49, *P* < 0.01, maternal predator odor exposure × sex interaction; Figure 5B). Female offspring from predator odor-exposed dams showed higher expression of *CRHR1* compared to female control offspring (*P* = 0.01). Overall, levels of predator-odor induced CORT (60 min time point, Figure 4) were positively correlated with the expression of *CRHR1* in the amygdala (*R*^2^ = 0.25, *P* = 0.01). There were no differences in the expression of *BDNF* in the amygdala. The expression of the other stress-related genes examined, *GR*, *MR*, *FKBP5*, *CRH*, and *CRHR2*, did not differ in the hippocampus or amygdala of offspring as a function of maternal predator odor exposure or offspring sex.

### DNA Methylation Analysis of *Bdnf* Exon *IV* in Adult Female Offspring

**Figure 6:**
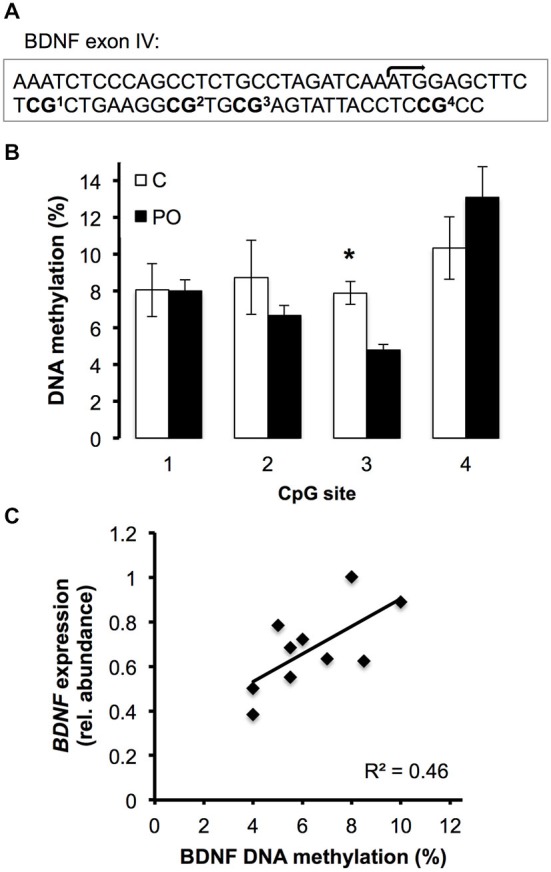
**DNA methyation level of the *Bdnf* exon *IV* in the hippocampus of female offspring of predator odor exposed (PO) and control (C) dams**. **(A)** Region sequenced with transcription initiation site (bent arrow) and CpG sites. **(B)** DNA methylation level (%) of the 4 sequenced CpG sites. **(C)** Correlation between the hippocampal *BDNF* expression and the hippocampal *Bdnf* exon *IV* CpG3 methylation level (%) (*P* = 0.03). Data are average ± SEM. *P ≤ 0.05, effect of maternal predator odor.

Due to the observed difference in *BDNF* expression in the hippocampus of female offspring from predator odor-exposed dams compared to controls and previous reports of altered epigenetic regulation of *Bdnf* exon *IV* as a function of early life environmental factors (Lubin et al., 2008; Roth et al., 2009), we examined DNA methylation levels in *Bdnf* exon *IV* in the hippocampus of adult female offspring (Figure 6A). Overall, levels of DNA methylation in female offspring differed significantly across CpG sites within *Bdnf* exon *IV* (*F*_(3,21)_ = 6.47, *P* = 0.003). In particular, CpG site 3 showed significantly lower methylation in female offspring of predator odor-exposed dams compared to controls (*t*_(10)_ = 4.09, *P* = 0.004; Figure 6B). Furthermore, hippocampal *BDNF* expression was positively correlated with hippocampal *Bdnf* exon *IV* CpG3 methylation level in females (*R*^2^ = 0.46, *P* = 0.03; Figure 6C). Methylation levels of other CpG sites within the exon did not differ significantly as a function of the mother’s odor exposure or vary in relation to *BDNF* expression (*P*’s > 0.05).

## Discussion

Exposure to predator threat plays a profound role in shaping ecosystems and communities (Love et al., 2013). In this study, we examined the stress-related phenotype in offspring from dams exposed to predator odor during the last half of pregnancy. Our hypotheses were that predator odor exposure would be linked to decreased body weight growth, increased anti-predator behaviors, heightened stress reactivity and altered neural gene regulation in key structures in the limbic system (hippocampus and amygdala) in adult offspring. We explored the possibility that alterations in offspring phenotypes may associate with differences in maternal care provided by their mothers within the first week of life. Offspring showed a sex-specific modulation of phenotype in response to maternal predator odor exposure. The expression of several stress-related genes was altered as a function of maternal predator odor exposure, with *Bdnf* showing site-specific epigenetic differences associated with transcriptional regulation of the *Bdnf* gene and maternal predator odor exposure.

### Maternal Behavior in Dams Exposed to Predator Odor During Pregnancy

Dams exposed to predator odor during pregnancy showed alterations in maternal behavior consisting of reduced licking of their pups on PN3. Decreased licking and grooming of pups has been linked to several indices of stress-related behavior in adult rat and mouse offspring (Weaver et al., 2004; Champagne et al., 2006). Therefore, the modulation of dam maternal behavior may reflect an adjustment of the females to a predator odor exposure itself or to cues provided by the pups as a function of the exposure. However, this transient decreased licking in predator odor exposed dams did not correlate with adult offspring phenotypic measures. We also did not detect changes in offspring body weight or growth associated with maternal predator odor exposure or maternal care. Future studies using a cross-fostering design would be important in determining the relative contribution of prenatal (dams) and postnatal (dams and pups) factors to differences in maternal care (e.g., Champagne et al., 2007).

### Behavioral and Endocrine Response to Predator Odor Exposure in Adult Offspring

The offspring of dams exposed to predator odor during pregnancy showed increased avoidance behavior to inescapable predator odor in adulthood compared to offspring from control dams. Female offspring from predator odor-exposed dams also showed decreased locomotor activity in the context of predator odor exposure in adulthood compared to control offspring. Compared to control female offspring, female offspring from predator odor-exposed dams showed a greater increase in CORT after exposure to predator odor in adulthood. Increased CORT levels after predator odor exposure adult mice have previously been linked to higher HPA axis reactivity and increased anxiety-like behaviors as well as with exposure to predator odor in early postnatal life in both sexes. Similarly, the offspring of gravid crickets exposed to live predator exhibit increased anti-predator behaviors (McAllister et al., 1999; Zheng and Quirion, 2004). Our data suggest an increased anti-predator behavior in adulthood in mice exposed to predator odor prenatally.

### Neural Gene Expression in Adult Offspring

The expression of stress-related genes was altered in offspring as a function of prenatal predator odor exposure. In the hippocampus, decreased expression of *BDNF* was observed in female offspring from predator odor-exposed dams. BDNF exon IV is the most abundant isoform of expressed BDNF transcript (Giannotti et al., 2014). During development (Andersen and Sonntag, 2014) and following stressors (Giannotti et al., 2014), the expression of total BDNF is closely related to the expression of BDNF exon IV mRNA. Notably, we observed changes in *BDNF* in female but not male offspring from predator odor-exposed dams relative to same-sex controls, together with enhanced CORT reactivity in female but not male offspring from predator odor-exposed dams. *BDNF* is a downstream target of an altered glucocorticoid signaling pathway, and its expression is altered in concert with dysregulated HPA function (Dwivedi et al., 2006; Lee and Sawa, 2014). There is evidence of decreased expression of *BDNF* in the hippocampus and amygdala of prenatally stressed animals (Lubin et al., 2008; Boersma et al., 2014). A decrease in hippocampus *BDNF* expression is also observed following chronic restraint stress (Bennett and Lagopoulos, 2014) and social defeat stress (Tsankova et al., 2006). Similar to our results, Taliaz et al. (2011) showed that lentivirus-induced reduction in hippocampal *BDNF* expression leads to prolonged elevation of circulating CORT. Early maternal separation, a potent method to induce impaired HPA responsiveness in later life, leads to long-term inhibition of BDNF expression in the hippocampus, coupled with impaired HPA response to forced swim (Roceri et al., 2004). Our results are thus consistent with the interpretation that decreased *BDNF* in female offspring of predator odor-exposed dams may result from altered stress-related signaling pathways linked to HPA function.

In further support of this interpretation, we found that *CRHR1* expression in the amygdala was increased among female offspring from predator odor-exposed dams but unchanged among males. *CRHR1* expression was also correlated with the increase in the levels of CORT during exposure to a predator odor. As *CRHR1* mediates the effects of *CRH*, an important activator of the HPA axis (Anisman, 2009), these results implicate altered *CRHR1* level in the increased CORT reactivity among female offspring from predator odor-exposed dams that were exposed to predator odor in adulthood. CRHR1 receptor is highly expressed in limbic areas such as the amygdala, including in the basolateral and the central nuclei, where it regulates the activation of stress-related responses (Joëls and Baram, 2009). These data support those of a recent study in a genetic model of anxiety-like behavior showing an association between higher levels of stress-induced CORT and increased *CRHR1* expression in male mice exposed to chronic mild stress (Zohar and Weinstock, 2011).

### *Bdnf* Exon *IV* Methylation and Transcriptional Regulation of *BDNF* in Adult Female Offspring

In adult female offspring from predator odor exposed dams, CpG3 within *Bdnf* exon *IV* showed significantly lower DNA methylation levels compared to control female offspring. Previous evidence has implicated DNA methylation of *Bdnf* exon *IV* in transcriptional regulation of the *BDNF* gene as a function of other forms of maternal adversity. For example, Roth et al. (2009) showed that stressed caregivers displaying increased maternal maltreatment showed lower *BDNF* transcript abundance in the medial pre-frontal cortex (mPFC) and higher DNA methylation levels of *Bdnf* exon *IV* in adult offspring. Several additional studies have also demonstrated the association between *BDNF* expression changes and alterations in DNA methylation, including in the context of early-life stress, though lower expression is not always associated with higher DNA methylation levels, and the specific CpG dinucleotides implicated in these effects vary in association with experimental parameters (reviewed in Boulle et al., 2012). In the context of maternal predator odor exposure in this study, *BDNF* transcript abundance in adult female offspring was positively correlated with levels of DNA methylation of *Bdnf* exon *IV* CpG 3, implicating this epigenetic modification in transcriptional regulation of the *BDNF* gene. The association between decreased exonic DNA methylation and decreased transcription are supported by a number of other studies, including the relationship between altered DNA methylation within exons and transcriptional regulation of the glucocorticoid receptor locus among adult offspring of mothers providing high compared to low levels of maternal care (McGowan et al., 2011). For example, exonic DNA methylation has been shown to associate with the transcriptional repression of retrotransposons and microRNAs within exonic regions as well as the regulation of cryptic alternative transcription start sites (Lev Maor et al., 2015). It is possible that hypermethylation of CpG site 3 in predator-odor exposed female offspring is associated with as-yet unrecognized regulatory control of BDNF exon IV. Future studies are required to examine this possibility, for example by targeted site-specific DNA methylation followed by chromatin immunprecipitation of RNA Polymerase associated with the BDNF exon IV or *in vitro* reporter assays (e.g., McGowan et al., 2009). Together, these results suggest an epigenetic mechanism underlying some long-term impacts of the prenatal exposure to predator odor.

## Conclusions

In this study, we found evidence for sex-specific effects of prenatal predator odor exposure on behavior, endocrine response and neural gene expression. Sex-specific alterations in behavior and neural gene expression appear to be a common feature of the impacts of early life adversity. For example, Mashoodh et al. (2009) found differential impacts of early postnatal exposure to predator odor in male and female offspring. Adult female offspring showed a less-anxious phenotype while males showed a more anxious phenotype in the open field. Coutellier and Würbel (2009) also found that the offspring of predator odor exposed mice dams showed reduced object recognition memory in female offspring. The opposite was observed in male offspring, indicating that increased maternal care might be able to rescue the adverse effect of early predator odor exposure in this model. Chronic variable stress in the third week of pregnancy in rats leads to increased anxiety-like behavior in the elevated-plus-maze (EPM) in young adult offspring and is associated with increased CRH and CRHR1 in the amygdala of female offspring and CRHR2 expression in offspring of both sexes (Zohar and Weinstock, 2011). Another study found that pregnant laboratory rats exposed to social defeat over 5 days in their last week of pregnancy produced adult offspring displaying greater HPA response to a restraint stress compared to control offspring (Brunton and Russell, 2010). Associated with this response, they found differential gene expression of the hippocampal MR and in the amygdala levels of GR and CRH in both sexes but decreased in hippocampal CA2 GR expression in female only. Thus, in these studies, female offspring exposed to a prenatal stress lead to broader impacts on neural gene expression in female than in male offspring. Responses to prenatal stressors may play both an adaptive role in stressful environments and lead to sex-specific differences in the risk for psychopathology, including an increased risk for affective disorders among females (Glover, 2011). Viewed from an evolutionary perspective, understanding the impacts of ethologically-relevant stressors such as predator odors may be important in elucidating mechanisms of plasticity associated with stress-related phenotypes.

The data reported here indicate that prenatal predator odor exposure alone, a psychological and ethologically-relevant stressor, is sufficient to alter offspring phenotype in a manner that persists to adulthood. We found that prenatal exposure to prenatal odor leads to phenotypic outcomes for offspring suggestive of an increased stress-related and defensive response. We also observed sex-specific differences in the impacts of prenatal predator odor exposure. To our knowledge, our study provides the first evidence of maternal transmission of behavioral sensitivity to predator odor, an unconditioned olfactory stimulus, and stress-related neural gene regulation through exposure to predator odor alone during pregnancy. Recently, an aversive association between a neutral odor (acetophenone) and mild foot shocks in the father was shown to lead to the retention of the aversive conditioning to this odor over multiple generations of mice offspring (Dias and Ressler, 2014). The potential influence of maternal predator odor exposure over multiple generations remains to be determined, though in several species multigenerational effects of predation exposure have been reported in the ecology literature (Sheriff et al., 2010; Love et al., 2013). Future studies using predator exposures in laboratory settings may help elucidate a mechanistic understanding of these effects.

## Conflict of Interest Statement

The authors declare that the research was conducted in the absence of any commercial or financial relationships that could be construed as a potential conflict of interest.

## Footnotes

^1^thepeemart.com

^2^www.ncbi.nlm.nih.gov

^3^useast.ensembl.org
